# Spec-RWKV: A Spectrum-Guided Multi-Scale Recurrent Modeling Framework for Multi-Center Resting-State fMRI-Assisted Diagnosis

**DOI:** 10.3390/brainsci16050455

**Published:** 2026-04-24

**Authors:** Sihang Peng, Qi Xu

**Affiliations:** College of Information Engineering, Shanghai Maritime University, Shanghai 201306, China; 202310310159@stu.shmtu.edu.cn

**Keywords:** resting-state fMRI, autism spectrum disorder, ADHD, multi-center data, recurrent neural network, spectral analysis

## Abstract

**Highlights:**

**What are the main findings?**

- Spec-RWKV showed competitive diagnostic performance on ABIDE-I and ADHD-200, with relatively consistent results under leave-one-site-out evaluation and simulated TR perturbations.
- Model-derived importance maps were concentrated in the default mode and frontoparietal networks, and ASD-related spectral differences were more evident in slower frequency bands.

**What are the implications of the main findings?**

- Defining temporal dynamics in physical time may help make multi-site rs-fMRI modeling less sensitive to acquisition differences in TR.
- Joint temporal–spectral modeling could provide cross-site analyses with intermediate patterns that remain easier to inspect and interpret.

**Abstract:**

Background: Multi-center resting-state functional magnetic resonance imaging (rs-fMRI) is important for neurodevelopmental disorder diagnosis, but cross-site differences in repetition time (TR) can cause temporal feature misalignment. In addition, blood-oxygenlevel-dependent (BOLD) signals are non-stationary, so disease-related information may be distributed across multiple time scales. Existing methods usually do not explicitly model physical sampling intervals or coordinate temporal and spectral information across scales, which may limit cross-site generalization in heterogeneous multi-center settings. Methods: We propose Spec-RWKV, a spectrum-guided linear recurrent framework for multi-site rs-fMRI diagnosis. It includes three components: PrismTimeMix, which models temporal dynamics using decay rates derived from physical half-lives and converts them adaptively across TRs; a TR-adaptive continuous wavelet transform, which aligns spectral representations across sites by adjusting frequency coverage; and spectrum-guided adaptive temporal aggregation, which uses spectral context to weight temporal features. Results: On ABIDE-I and ADHD-200, Spec-RWKV achieved AUCs of 75.86% and 76.31%, respectively. Under leave-one-site-out validation, it achieved the best mean AUC on ABIDE-I and the best mean accuracy and AUC on ADHD-200. Conclusions: Spec-RWKV explicitly models sampling-rate differences and multi-scale spectral structure, with results supporting strong cross-site generalizability.

## 1. Introduction

Resting-state functional magnetic resonance imaging (rs-fMRI) measures spontaneous brain activity—that is, activity occurring naturally rather than in response to tasks. It relies on blood-oxygen-level-dependent (BOLD) signals, which reflect changes in blood oxygenation as a proxy for neural activity [1]. Neuroimaging biomarkers derived from rs-fMRI—measurable brain signatures associated with a clinical condition—show promise for neurodevelopmental disorders such as autism spectrum disorder (ASD) and attention-deficit/hyperactivity disorder (ADHD) [2]. However, single-site studies are often limited in sample size and demographic diversity. This makes it difficult to establish reliable and generalizable biomarkers. Multi-site datasets, such as ABIDE [3] and ADHD-200 [4], address this by pooling data across institutions. Larger and more diverse samples provide a more realistic test bed for evaluating generalizability. Nevertheless, multi-site data introduces substantial variability in scanners and acquisition protocols. When sample sizes are small and the disease signal is weak relative to acquisition-related variance, models are prone to learning spurious correlations—capturing patterns tied to equipment or protocol differences rather than to the disorder itself [5,6,7].

Among these sources of heterogeneity, variation in repetition time (TR)—the interval between successive volume acquisitions—directly affects temporal modeling. When TR differs across sites, the same number of time steps spans different physical durations. Discrete-step temporal features thus become physiologically inconsistent across cohorts. Different TRs correspond to different sampling rates and, consequently, different Nyquist frequencies. Sites with lower sampling rates may under-sample high-frequency physiological signals, causing cardiac and respiratory rhythms to alias into the BOLD signal in site-dependent ways. This can systematically alter commonly used metrics, including functional connectivity strength and low-frequency oscillation amplitude [8,9]. The problem is compounded by the non-stationary nature of BOLD signals [10]. Disease-related information may appear as brief transient events lasting a few seconds, or as slower shifts spanning tens of seconds. Capturing both requires modeling at multiple temporal scales. When temporal scales are defined in discrete time steps rather than physical duration, the same scale index corresponds to different physiological windows across sites, making multi-scale modeling inconsistent.

Existing methods address these challenges only partially. The most common strategy for handling TR differences is to resample all signals to a uniform temporal resolution during preprocessing [11]. However, interpolation can introduce artifacts and alter the autocorrelation structure of the signal [8]. Resampling can also change the spectral aliasing profile and redistribute power across low-frequency bands [9]. These preprocessing strategies, therefore, do not fully resolve the underlying inconsistency in physical time scales. More recent approaches introduce time awareness through neural ordinary differential equations [12] or continuous-time positional encoding [13]. These methods address part of the problem, but they share a common limitation: TR information is typically injected only at the input stage and does not propagate into the recurrent dynamics that accumulate temporal evidence. Recurrent models that apply attention over sliding-window dynamic functional connectivity sequences [14] and prompt-based frameworks that augment graph networks with multi-level knowledge [15] have achieved competitive results on multi-site benchmarks, yet neither explicitly conditions its temporal or spectral representations on the acquisition TR.

A related gap exists in multi-scale and spectral modeling. Fixed-window methods segment data into a fixed number of time steps, so the same window spans different physical durations at different TRs. Wavelet-based methods with uniform decomposition scales ignore the fact that the meaningful frequency range depends on the sampling rate, which varies with TR. Most existing fusion strategies combine spectral and temporal features only at a late stage, so the two branches cannot inform each other during representation learning.

Continuous-time modeling, adaptive spectral analysis, and cross-modal fusion have each been explored separately. However, we are not aware of a framework that jointly addresses these aspects. In particular, TR heterogeneity is rarely treated as an explicit constraint on both temporal and spectral modeling. To address this, we propose Spec-RWKV for multi-site rs-fMRI diagnosis. The framework combines three core components: TR-aware temporal modeling, sampling-rate-adaptive spectral analysis, and spectrum-guided temporal aggregation.

At its core, Spec-RWKV builds on Receptance Weighted Key Value (RWKV) [16], a linear-complexity recurrent attention mechanism. Standard self-attention compares all time-step pairs and scales quadratically with sequence length. RWKV instead accumulates information incrementally at linear cost. Building on this backbone, we introduce a multi-scale decay mechanism called PrismTimeMix. Each decay rate in PrismTimeMix corresponds to a specific BOLD frequency band and is defined as a physical half-life measured in seconds. Crucially, these decay rates are then converted to per-step parameters using each sample’s actual TR, so the same physical time scale is preserved regardless of how densely the signal was sampled. This means TR adaptation is no longer confined to input embeddings or positional encoding. Instead, it directly shapes how the model accumulates and forgets information over time. On the spectral side, we introduce a TR-adaptive continuous wavelet transform (CWT). CWT decomposes a signal into frequency components at each time point. Unlike fixed-scale decomposition, our variant adjusts its frequency coverage and scale resolution per sample to match each site’s actual sampling rate, keeping frequency representations aligned across sites. Finally, we introduce a pooling module that uses spectral features to reweight temporal information across time steps. We call this module Spectrum-guided Adaptive Temporal Aggregation (SATA). Rather than keeping frequency and temporal branches independent until a final fusion step, this design lets spectral context directly determine which time steps matter most for prediction.

The main contributions of this work are summarized as follows:

1. We propose Spec-RWKV, a linear recurrent framework in which each temporal decay rate corresponds to a specific BOLD oscillatory band. The decay rates are defined as physical half-lives in seconds and converted to per-step values using each sample’s TR. This design embeds TR adaptation directly into the recurrent dynamics, ensuring that the model operates in consistent physical time units across sites with different TRs.
2. We design a frequency-temporal coordination mechanism in which TR-adaptive spectral features serve two roles: aligning frequency-domain representations across sites, and guiding temporal aggregation weights. This creates a direct information flow from the spectral branch to the temporal branch.
3. On ABIDE-I and ADHD-200, Spec-RWKV achieves the area under the receiver operating characteristic curve (AUC) values of 75.86% and 76.31%, respectively. Under leave-one-site-out evaluation, it attains the best aggregate AUC on ABIDE-I and the best aggregate accuracy and AUC on ADHD-200 among the evaluated methods, indicating competitive cross-site robustness under this stricter setting.

The remainder of this paper is organized as follows: Section 2 introduces data and methods; Section 3 presents experimental results and ablation analysis; Section 4 discusses mechanistic interpretation and limitations; Section 5 concludes the paper.

## 2. Materials and Methods

### 2.1. Datasets

#### 2.1.1. ABIDE-I

ABIDE-I [3] is a multi-site open-access dataset from the International Neuroimaging Data-sharing Initiative (INDI), comprising rs-fMRI, T1 structural images, and phenotypic data from 1112 individuals across 20 international sites (539 ASD, 573 typically developing controls; ages 6–64, mean ≈17 years). We use preprocessed data from the ABIDE Preprocessed Connectomes Project (PCP) with the CPAC pipeline, without band-pass filtering or global signal regression. Subjects were excluded if any ROI had a constant (all-zero) time series. After quality control, 871 subjects (403 ASD/468 controls) remained, covering all 20 sites (Table 1).

The dataset exhibits substantial heterogeneity. Site-level sample sizes range from 11 to 172, and class balance varies considerably (e.g., USM has 43 ASD vs. 24 controls, while UM_1 has 34 ASD vs. 52 controls). TR values range from 1.5 to 3.0 s across sites, reflecting diverse acquisition protocols. This variation poses a key challenge for cross-site generalization, motivating the protocol-invariant design of our method.

#### 2.1.2. ADHD-200

ADHD-200 [4] is a multi-site dataset released for the 2011 ADHD-200 Global Competition, containing rs-fMRI, structural MRI, and phenotypic information from 776 individuals across 9 sites. The original labels distinguish three ADHD subtypes (Combined, Hyperactive/Impulsive, and Inattentive); we merge all subtypes into a single ADHD group for binary classification. After quality control, 635 subjects from 9 sites remain. Pittsburgh and WashU contain only control subjects, which affects how the leave-one-site-out evaluation is designed for these sites. Site-wise sample statistics are reported in Table 2.

**Figure 1:**
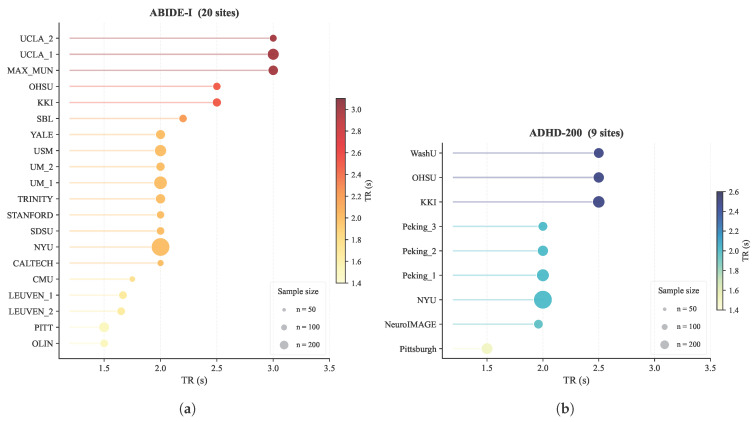
Cross-site TR heterogeneity in the two benchmark datasets. (**a**) ABIDE-I: TR ranges from 1.5 to 3.0 across 20 sites. (**b**) ADHD-200: TR ranges from 1.5 to 2.5 s across 9 sites, with less variation than ABIDE-I.

Both datasets pool data from multiple sites with considerable differences in acquisition protocols—including TR, field strength, pulse sequence, and spatial resolution [17]. These variations affect the BOLD signal’s spatiotemporal properties and introduce systematic site differences. Among protocol factors, TR is especially critical: it sets the BOLD sampling rate and the effective Nyquist limit, so TR differences change how physiological noise aliases into the data and influence frequency-sensitive rs-fMRI measures [9]. Broader hardware and protocol effects can confound models and limit generalization to new sites [7]. We therefore treat TR variation as a key design constraint, combining protocol-aware preprocessing with protocol-invariant modeling to minimize cross-site mismatch. Figure 1 shows that TR spans 1.5–3.0 s in ABIDE-I and is more narrowly distributed (1.5–2.5 s) in ADHD-200.

### 2.2. Data Preprocessing

For the ABIDE-I and ADHD-200 cohorts, we constructed atlas-based region-of-interest (ROI) representations using two parcellation schemes: the Schaefer 7-network atlas (400 ROIs) [18] and the AAL atlas (116 ROIs) [19]. The Schaefer atlas divides the cortex functionally, aligned with seven canonical brain networks [20]. The AAL atlas uses anatomical boundaries instead. Using both atlases allows us to test whether findings hold across different parcellation scales and definitions.

Under each atlas, we represent each subject’s rs-fMRI data as a time-series matrix $X\in R^{T\times N}$, where *N* is the number of ROIs and *T* is the sequence length. We z-score normalize each ROI time series independently to reduce amplitude differences across subjects and sites:

(1) $${\tilde{x}}_{n,t}=\frac{x_{n,t}-\mu_{n}}{\sigma_{n}+\epsilon },$$

where $\mu_{n}$ and $\sigma_{n}$ are the mean and standard deviation of the *n*-th ROI time series, and ϵ is a small constant for numerical stability. Subjects with any constant-valued ROI (zero variance) are excluded.

### 2.3. Spec-RWKV Framework

#### 2.3.1. Overall Architecture

**Figure 2:**
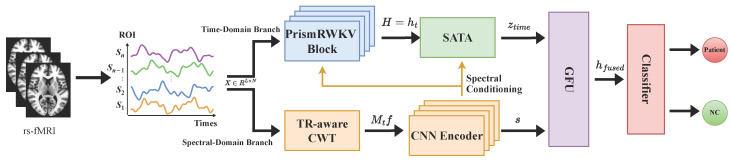
Overview of the Spec-RWKV framework. The lower spectral pathway computes a TR-adaptive spectral context vector s in physical frequency units, which conditions the upper temporal backbone at two points: by modulating multi-scale decay rates in PrismTimeMix and by constructing the aggregation query in SATA. The temporal pathway processes ROI time series with stacked PrismRWKV blocks whose decay rates are defined in physical time (seconds). The two pathways are merged by a Gated Fusion Unit (GFU) for binary classification.

Spec-RWKV uses a dual-pathway architecture with a temporal backbone and a spectral branch (Figure 2). Given an ROI time series segment $X\in R^{L\times N}$, the model projects each ROI’s features into a shared hidden space, forming token representations. The temporal backbone models dependencies across multiple time scales. Meanwhile, the spectral branch extracts a spectral context vector whose frequency axes are aligned to physical units (Hz) using each sample’s TR.

The spectral context vector s is computed once per segment and conditions the temporal pathway at two points. First, it modulates the multi-scale decay rates in PrismTimeMix. This controls how quickly past information decays, allowing memory dynamics to adapt to each sample’s spectral profile. Second, it constructs the query in Spectrum-guided Adaptive Temporal Aggregation (SATA), guiding how temporal hidden states are aggregated.

For long sequences, Spec-RWKV operates on fixed-length segments, producing segment-level predictions. These are aggregated to subject-level decisions by averaging segment logits. During training, segments are selected stochastically to improve robustness. At inference, selection is deterministic for stable evaluation. The following subsections describe each component in detail.

#### 2.3.2. Temporal Backbone: RWKV Recurrent Attention and PrismTimeMix

The temporal backbone consists of $L_{b}$ stacked PrismRWKV blocks. Each block uses a pre-LayerNorm residual structure (normalization applied before each main operation):

(2) $$h^{\left(\ell \right)}=h^{\left(\ell -1\right)}+\mathrm{TimeMix}\left(\mathrm{LN}(h^{\left(\ell -1\right)})\right),\;h^{\left(\ell \right)}=h^{\left(\ell \right)}+\mathrm{ChannelMix}\left(\mathrm{LN}(h^{\left(\ell \right)})\right).$$

**Figure 3:**
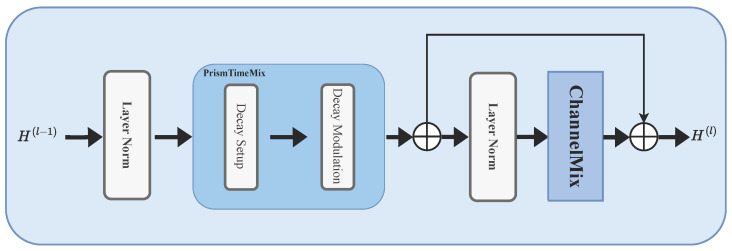
Internal structure of a single PrismRWKV block. Each block consists of a multi-scale TimeMix module (PrismTimeMix) and a ChannelMix module, connected through pre-LayerNorm residual paths. PrismTimeMix replaces the standard single-decay recurrent attention with *K* parallel scale heads, each operating at a different physical time constant. The spectral context vector s enters at the TimeMix stage to modulate decay rates.

Here $h^{\left(\ell \right)}$ is the hidden state at layer *ℓ*. We use RWKV as the temporal backbone because it processes time series with linear complexity, making it efficient for moderately long sequences with limited training samples. Figure 3 shows the internal structure of a single PrismRWKV block.

Each PrismRWKV block separates temporal mixing from feature mixing through two residual stages. This is similar to standard Transformer blocks, but uses linear-complexity recurrence instead of self-attention. The first stage applies PrismTimeMix to model temporal dependencies. The second applies ChannelMix (a gated feedforward layer) to combine information across features.

Standard RWKV uses a single decay rate, which limits its ability to capture both fast transients and slow drifts in BOLD signals. PrismTimeMix addresses this by maintaining multiple parallel memory channels. Each channel forgets information at a rate tied to a specific BOLD frequency band (e.g., slow-5 vs. slow-4). Crucially, these decay rates are defined in seconds rather than in discrete time steps. This ensures that the same physical memory span is preserved regardless of TR.

Formally, PrismTimeMix maintains *K* parallel scale heads, each associated with a physical half-life $\tau_{k}$ (seconds). The corresponding per-second and per-step decay rates are defined as

(3) $$\alpha_{k}^{\left(\sec\right)}=2^{-1/\tau_{k}},\;\alpha_{k}^{\left(\mathrm{step}\right)}={\left(\alpha_{k}^{\left(\sec\right)}\right)}^{\mathrm{TR}},$$

where TR is the sampling interval (in seconds) of the current sample. This conversion ensures that each scale head maintains the same physical time constant across sites, regardless of sampling rate.

**Figure 4:**
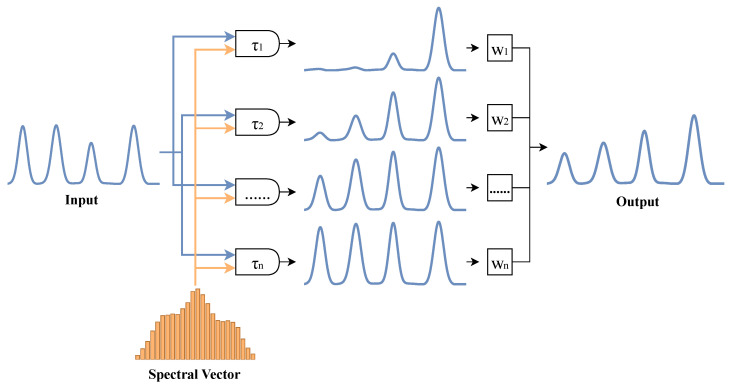
Multi-scale decay mechanism in PrismTimeMix. Each of *K* parallel heads operates at a physical half-life $\tau_{k}$ tied to a BOLD frequency band (e.g., slow-5 through slow-2). Decay rates are converted from per-second to per-step values using the sample’s TR, preserving consistent physical memory spans across sites. The spectral context vector s further modulates these per-sample rates. Blue arrows indicate the main signal flow; orange arrows denote spectral context modulation from s.

The spectral context vector s further modulates these decay rates, allowing temporal memory to adapt to each sample’s spectral characteristics (Figure 4):

(4) $$\alpha_{k}^{\left(\sec,\mathrm{eff}\right)}=\sigma \;\left({\mathrm{logit}}_{k}+\gamma \cdot \tanh\;\left({\left[W_{r}\;\mathrm{LN}\left(W_{p}s\right)\right]}_{k}\right)\right),\;k=1,\ldots ,K,$$

(5) $$\alpha_{k}^{\left(\mathrm{step},\mathrm{eff}\right)}={\left(\alpha_{k}^{\left(\sec,\mathrm{eff}\right)}\right)}^{\mathrm{TR}},\;w_{k}=\log\;\left(-\log(\alpha_{k}^{\left(\mathrm{step},\mathrm{eff}\right)})\right).$$

Each scale head then performs recurrent attention using its decay parameter $w_{k}$. The resulting responses are fused across scales. The key point is that spectral information shapes the temporal memory window at every recurrent step, not just at input or output.

Without explicit constraints, gradient-based optimization may concentrate all weights on a single scale head. This would reduce PrismTimeMix to a single-scale model. To prevent this, we apply three auxiliary losses during training. Let $g_{b,k}$ denote the normalized mixing weight of the *k*-th scale head for the *b*-th sample. The entropy term is defined as

(6) $$L_{\mathrm{ent}}=-\frac{1}{B}\sum_{b=1}^{B}\sum_{k=1}^{K}g_{b,k}\log\left(g_{b,k}+\epsilon \right),$$

which discourages premature concentration on a single scale head. In addition, we compute the fraction of spectral energy below 0.08 Hz to encourage the model to assign more weight to longer-timescale heads when low-frequency activity dominates:

(7) $$r_{\mathrm{low}}=\frac{\sum_{s:\;f(s)\leq 0.08\;\mathrm{Hz}}E_{s}}{\sum_{s}E_{s}+\varepsilon },\;L_{\mathrm{guide}}=\mathrm{MSE}\left(\bar{g},\;r_{\mathrm{low}}\right),$$

where $E_{s}$ denotes spectral energy at scale *s* and $\bar{g}$ is the average weight assigned to the longer-timescale heads. Finally, a subsequence consistency term ensures that predictions from the full sequence and from randomly sampled subsequences agree.

#### 2.3.3. Spectral Branch and Spectrum-Guided Temporal Aggregation

The spectral branch aligns time–frequency representations from different sites to a common physical frequency scale. Because BOLD signals are non-stationary, static spectral summaries are insufficient to characterize their time-varying oscillatory patterns. We therefore use the continuous wavelet transform (CWT), which provides a joint time–frequency representation [21,22]. CWT preserves both frequency and temporal information, making it suitable for non-stationary BOLD signals.

Rather than using fixed-frequency indices, the spectral branch adapts its frequency coverage to each sample’s TR. This reduces protocol-induced spectral mismatch across sites. Let $r_{\mathrm{TR}}={\mathrm{TR}}_{\mathrm{base}}/{\mathrm{TR}}_{\mathrm{current}}$ denote the ratio between a reference TR and the current sample’s TR. The adaptive upper frequency limit and number of CWT scales are then

(8) $$f_{\max}^{\mathrm{adapt}}=\min\;\left(f_{\max}^{\mathrm{base}}\cdot r_{\mathrm{TR}},\;0.3\right),\;n_{\mathrm{scales}}^{\mathrm{adapt}}=\min\;\left(\left\lfloor n_{\mathrm{scales}}^{\mathrm{base}}\cdot r_{\mathrm{TR}}\right\rfloor ,\;12\right).$$

**Figure 5:**
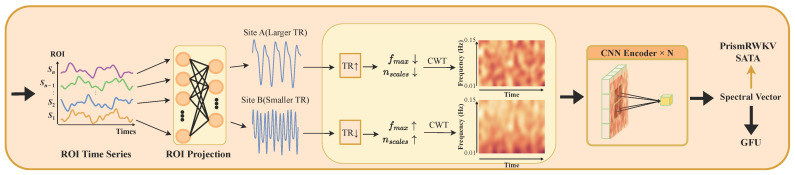
Architecture of the TR-adaptive spectral branch. The input ROI time series is transformed by a CWT whose frequency range and scale count are adjusted to the sample’s TR (see Figure 6 for the alignment effect). Arrows indicate the direction of change: a longer TR (TR↑) reduces both the maximum frequency $f_{\max}$ and the number of scales $n_{\mathrm{scales}}$ (↓), and vice versa. A frequency encoder then compresses the time–frequency map into a spectral context vector s, which conditions both PrismTimeMix decay rates and the SATA aggregation query.

**Figure 6:**
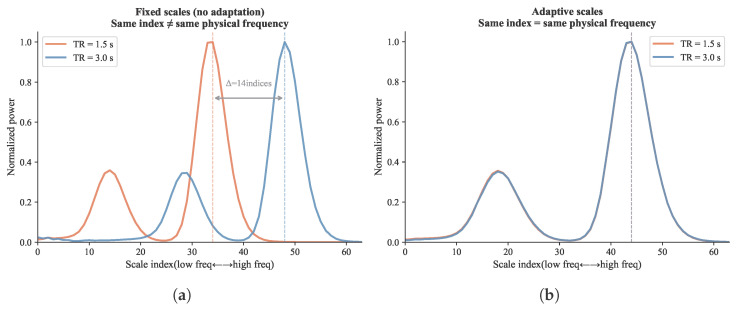
Effect of TR-adaptive scale selection on cross-site spectral alignment. (**a**) Fixed-scale CWT: when the same scale indices are applied to samples with different TRs, each index maps to a different physical frequency, creating systematic spectral mismatch across sites. (**b**) TR-adaptive CWT: scale parameters are adjusted per sample so that each scale index corresponds to approximately the same physical frequency regardless of TR. This alignment is essential for the spectral context vector to carry comparable information across sites.

In practical terms, a site with TR = 1.5 s can resolve higher frequencies than a site with TR = 3.0 s. The adaptive mechanism accounts for this. When the sampling rate is higher, it widens the frequency range and adds finer scales. When the rate is lower, it narrows the range and uses coarser scales. This means that each scale index approximately corresponds to the same physical frequency across sites. Figure 5 and Figure 6 show the complete processing pipeline and the resulting frequency alignment.

At the aggregation stage, we introduce Spectrum-guided Adaptive Temporal Aggregation (SATA), a pooling module that uses spectral context to combine temporal hidden states across time steps. The intuition is that not all time steps in a resting-state scan are equally informative. Some may capture transient neural events relevant to the disorder, while others may be dominated by noise or motion. SATA lets the spectral branch determine which time steps to emphasize. Formally,

(9) $$q=W_{q}\left(s+\beta \;g_{f}\right),\;k_{t}=W_{k}h_{t},$$

(10) $$\alpha =\mathrm{softmax}\;\left(\frac{Kq}{\sqrt{D_{s}}}\right)\in \Delta^{L},\;z_{\mathrm{time}}=\sum_{t=1}^{L}\alpha_{t}\;h_{t},$$

where $K={\left[k_{1},\ldots ,k_{L}\right]}^{⊤}$ and $D_{s}$ is the query/key dimension. Here $g_{f}$ is an auxiliary spectral summary projected to the query space, biasing aggregation toward spectrally salient time steps. In effect, SATA upweights time steps whose temporal content is most consistent with the dominant spectral cues.

**Figure 7:**
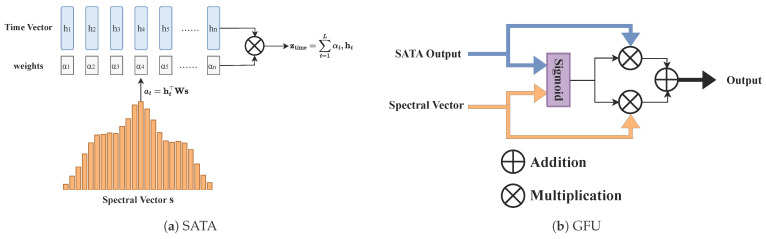
(**a**) Spectrum-guided Adaptive Temporal Aggregation (SATA). The spectral context vector s is projected into a query, while each temporal hidden state $h_{t}$ is projected into a key. The query–key similarity determines how much each time step contributes to the aggregated representation; time steps whose content is most consistent with the dominant spectral cues receive higher weight. (**b**) Gated Fusion Unit (GFU). The temporal representation and spectral context are combined through learned element-wise gates, allowing the model to adaptively balance temporal and spectral contributions per feature dimension.

The temporal representation $z_{\mathrm{time}}$ and spectral context s are then combined through a Gated Fusion Unit (GFU):

(11) $$z_{g}=\sigma \left(W_{1}z_{\mathrm{time}}+W_{2}s\right),\;h_{\mathrm{fused}}=\mathrm{LN}\left(z_{g}⊙z_{\mathrm{time}}+\left(1-z_{g}\right)⊙s\right),$$

where $z_{g}$ is the element-wise gating weight. Each feature dimension independently balances temporal and spectral contributions, allowing the model to emphasize whichever source is more informative. Figure 7 illustrates the mechanisms of SATA and GFU.

#### 2.3.4. Training Objectives and Optimization Strategy

The training objective combines a classification loss with auxiliary regularization terms. For classification, we use Asymmetric Focal Loss (AFL) [23], which applies different focusing parameters to positive and negative samples, helping the model attend to the minority class without aggressive resampling. The overall objective is

(12) $$L=L_{\mathrm{AFL}}+\lambda_{\mathrm{ent}}L_{\mathrm{ent}}+\lambda_{\mathrm{guide}}L_{\mathrm{guide}}+\lambda_{\mathrm{cons}}L_{\mathrm{cons}}.$$

$L_{\mathrm{ent}}$, $L_{\mathrm{guide}}$, and $L_{\mathrm{cons}}$ encourage scale diversity, spectrum–memory alignment, and consistency between full-sequence and subsequence predictions, respectively. Specifically, $L_{\mathrm{cons}}$ encourages two randomly sampled subsequences from the same subject to produce similar latent representations, implemented as $L_{\mathrm{cons}}=1-\cos\left(z_{1},z_{2}\right)$. Optimization uses the Adam optimizer with OneCycle learning-rate scheduling [24].

## 3. Results

### 3.1. Experimental Settings and Evaluation Metrics

#### 3.1.1. Experimental Settings

We evaluated diagnostic performance under three protocols: mixed-site 10-fold cross-validation, leave-one-site-out (LOSO) testing, in which one site is held out entirely for testing and the model is trained on all remaining sites, and cross-atlas validation.

In the mixed-site protocol, samples from all sites were pooled and evaluated with stratified 10-fold cross-validation. ABIDE-I included 871 subjects from 20 sites, and ADHD-200 included 635 subjects from nine sites. Each experiment was repeated with five random seeds. The reported results are the mean and standard deviation over 50 test runs.

In the LOSO protocol, one site was held out for testing, and the remaining sites were used for training under the Schaefer atlas. Results were then aggregated across all held-out sites. In the cross-atlas protocol, the mixed-site evaluation was repeated on the AAL atlas with 116 ROIs.

Each fold was trained for 20 epochs without early stopping, and the final checkpoint was used for testing. We optimized the model with Adam and a OneCycle schedule. The maximum learning rate was $10^{-4}$, the initial learning rate was $4\times 10^{-6}$, and the final learning rate was $10^{-8}$. The warm-up phase accounted for the first 30% of the training steps. We set gradient clipping to 1.0, weight decay to $10^{-3}$, and batch size to 32.

During training, we randomly sampled up to two segments of length 64 from each subject. During inference, we evaluated up to six evenly spaced segments with a stride of 48 and averaged their logits for subject-level prediction. The classification loss was AFL, with α=0.25, $\gamma_{+}=1.0$, and $\gamma_{-}=2.0$.

In the TR-adaptive CWT module, we set the reference repetition time to ${\mathrm{TR}}_{\mathrm{base}}=2.0\;s$. This is the most common TR value in both datasets. We set the base upper frequency to $f_{\max}^{\mathrm{base}}=0.15\;\mathrm{Hz}$ and the base number of scales to $n_{\mathrm{scales}}^{\mathrm{base}}=6$.

PrismTimeMix used K=4 parallel time-scale heads with physical half-lives $\tau_{k}\in \left\{2,8,32,128\right\}\;s$. These heads cover both short- and long-range temporal dependencies in the BOLD signal. The spectral modulation strength γ was implemented as a learnable scalar and initialized to zero.

In SATA, we fixed the spectral guidance coefficient at β=0.1. This coefficient controls how strongly the auxiliary spectral summary influences the aggregation query. The auxiliary loss weights were set to $\lambda_{\mathrm{ent}}=10^{-3}$, $\lambda_{\mathrm{guide}}=10^{-3}$, and $\lambda_{\mathrm{cons}}=0.5$.

We did not apply over-sampling or under-sampling, including on ADHD-200, where class imbalance was more pronounced. In the LOSO evaluation, we preserved the native site-level class distribution. This includes the Pittsburgh and WashU sites, which contained only control participants after quality control. We handled class imbalance at the loss level rather than by rebalancing the data distribution.

To characterize cross-site behavior more fully, we conducted three supplementary analyses. First, we examined per-site LOSO performance on ABIDE-I, summarized with balanced accuracy (BA) and AUC. Second, we tested whether frozen model embeddings retain site-discriminative information. We used a linear classifier with 5-fold stratified cross-validation and permutation testing. Third, we evaluated robustness to simulated TR changes by resampling time series to target TR values with piecewise linear interpolation.

All experiments were conducted on a single NVIDIA RTX 4080 Super. The model has approximately 12.3M parameters. Single-epoch training takes about 45 s on ABIDE-I and 32 s on ADHD-200. Inference averages approximately 18 ms per sample, with peak memory usage of about 6.2 GB.

#### 3.1.2. Evaluation Metrics

We evaluate model performance with four binary classification metrics: accuracy (ACC), precision, recall, and AUC. Here, TP, TN, FP, and FN denote the counts of true positives, true negatives, false positives, and false negatives, respectively.

(13) $$\mathrm{Accuracy}=\frac{TP+TN}{TP+TN+FP+FN}$$

(14) $$\mathrm{Precision}=\frac{TP}{TP+FP}$$

(15) $$\mathrm{Recall}=\frac{TP}{TP+FN}$$

The area under the receiver operating characteristic curve (AUC),which summarizes how well the model separates the two classes across all possible decision thresholds, measures how well the model discriminates between classes across all decision thresholds. All metrics are reported as mean ± standard deviation over 50 test runs. Statistical significance was assessed with paired *t*-tests at a threshold of p<0.05.

### 3.2. Baseline Methods

We compared Spec-RWKV with representative baselines from five groups. The traditional baseline was SVM, which used statistical features derived from ROI time series. The recurrent baselines were LSTM [25], CNN-LSTM, and AttnLSTM [14]. AttnLSTM models temporal dependencies in functional-connectivity sequences using attention and LSTM modules. The graph-based baselines were BrainNetCNN [26], PLSNet [27], and MAHGCN [28]. The Transformer-style baselines were SwinT [29], BolT [11], STARFormer [30], and Com-BrainTF [31]. Finally, BrainPrompt [15] is a prompt-based model designed for multi-site neuroimaging classification. Spec-RWKV and BolT operate directly on raw ROI time series. BrainPrompt, AttnLSTM, BrainNetCNN, and SVM rely on functional connectivity or other derived representations. Together, these baselines cover complementary modeling strategies for multi-site rs-fMRI classification.

### 3.3. Experimental Results

#### 3.3.1. Comparison with Mainstream Methods

Table 3 reports the main comparison on the Schaefer atlas under mixed-site 10-fold cross-validation. On ABIDE-I, Spec-RWKV achieved the best AUC (75.86%), precision (74.97%), and accuracy (73.27%). BrainPrompt, AttnLSTM, BolT, and PLSNet all remained competitive but stayed below Spec-RWKV in AUC. This advantage held across all baseline families, suggesting that the gain is not specific to any single modeling strategy.

On ADHD-200, Spec-RWKV again achieved the best overall performance, with 72.87% accuracy, 74.64% precision, and 76.31% AUC. BrainPrompt and AttnLSTM were both competitive but did not exceed Spec-RWKV in AUC. ADHD-200 has a narrower TR range than ABIDE-I, so the margin attributable to TR-aware modeling may be smaller on this dataset.

Table 4 reports the corresponding results on the AAL atlas. Spec-RWKV again achieved the best overall performance on both datasets. This indicates that the advantage is not tied to a single parcellation scheme.

The LOSO results (Table 5) evaluate a more challenging scenario: the model must generalize to an entirely unseen acquisition site. On ABIDE-I, Spec-RWKV achieved 72.78% accuracy and 75.86% AUC, which was the best aggregate AUC among the evaluated methods and slightly higher than BrainPrompt on both accuracy and AUC. The gap relative to BolT was larger (+5.39 in accuracy and +5.62 in AUC), and BolT does not include explicit TR adaptation.

In mixed-site cross-validation, every site contributes training data, so models may partly learn site-specific patterns. In LOSO, the held-out site is entirely new, and models that have learned such patterns may generalize less well. The overall pattern under LOSO is consistent with the possibility that TR-aware modeling reduces reliance on site-specific variation, although other factors may also contribute.

On ADHD-200, LOSO performance dropped for all methods. This is expected because the dataset has fewer sites and a stronger class imbalance. Pittsburgh and WashU contain only controls, so holding out either site removes a substantial fraction of negative examples from the training set. Even under these conditions, Spec-RWKV achieved the best LOSO accuracy and AUC.

#### 3.3.2. Per-Site Leave-One-Site-Out Performance

We further examined LOSO performance across the 20 held-out ABIDE-I sites. Table 6 reports the mean, standard deviation, and interquartile range (IQR) of balanced accuracy (BA) and AUC across sites, and Table A1 in Appendix A provides the full site-wise results.

Spec-RWKV achieved the highest mean BA with one of the tightest site-level spreads. BolT was competitive in mean AUC but showed a broader, lower BA distribution. BrainPrompt showed improved site-level stability. AttnLSTM exhibited a broader spread than the top performers.

**Figure 8:**
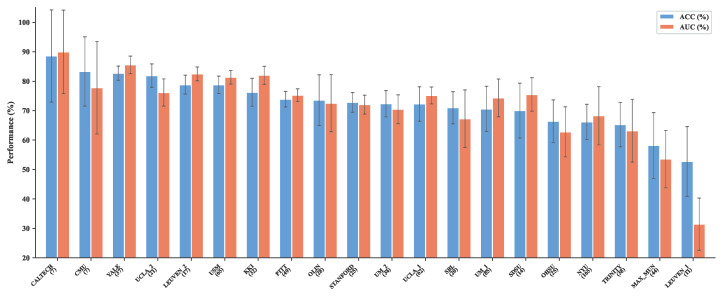
Per-site LOSO performance of Spec-RWKV on ABIDE-I. Each pair of bars shows the accuracy (blue) and AUC (coral) when that site is held out as the test set. Error bars denote standard deviation across five random seeds. Sites are sorted by descending accuracy; parenthesized numbers indicate site sample size.

To visualize site-level variability, Figure 8 presents the per-site accuracy and AUC of Spec-RWKV across all 20 held-out ABIDE-I sites. Performance is generally stable for larger sites (e.g., NYU, UM_1, USM), whereas smaller sites exhibit wider confidence intervals. A few sites with atypical class ratios or scanner protocols (e.g., LEUVEN_1, MAX_MUN) show notably lower performance, consistent with the challenges identified in Table 6.

#### 3.3.3. Site Prediction Analysis

We evaluated site information in the learned representations using a 20-way site-prediction task on the ABIDE-I dataset. Each trained model was frozen, and its penultimate representations were extracted for all 871 subjects. A linear classifier was then trained to predict the acquisition site, following the protocol described in Section 3.1.

Table 7 reports balanced accuracy for 20-way site prediction (chance = 5%). AttnLSTM achieved the lowest BA at 8.23%, followed by Spec-RWKV at 12.38%. BolT and BrainPrompt showed substantially higher values. This pattern suggests that Spec-RWKV embeddings retain less site-specific information than those of BolT and BrainPrompt. Combined with its stronger diagnostic performance, the result is consistent with reduced reliance on site-related variation, although it does not by itself identify which disorder-relevant features are being captured.

#### 3.3.4. TR Robustness Analysis

To test whether model outputs remain stable when the temporal resolution changes, we resampled each ABIDE-I time series to simulated TR values of 1.5, 2.0, 2.5, 3.0, and 4.0 s using piecewise linear interpolation and re-ran inference with the trained models. Because this experiment measures output consistency under controlled resampling rather than diagnostic generalization, all 871 subjects were included regardless of their role during training. The absolute AUC values in Table 8 are therefore not directly comparable to the held-out cross-validation results in Table 3; the quantity of interest is the relative variation across TR conditions.

Table 8 shows that Spec-RWKV maintained the most consistent outputs across simulated TR values, with the smallest fluctuation range (ΔAUC=0.011). AttnLSTM was comparatively stable and consistently outperformed BrainPrompt, which showed a larger drop around TR=3.0s.

#### 3.3.5. Ablation Studies

We performed ablation experiments on ABIDE-I and ADHD-200 to examine the contribution of the main temporal–spectral components. We removed PrismTimeMix, the CWT branch, and SATA either individually or jointly. We also evaluated two mechanism variants. No-TR-Adapt replaces TR-adaptive frequency scaling and decay conversion with fixed base-TR behavior. No-Spec-Guide disables spectral-conditioned modulation and spectral-guided aggregation while preserving the recurrent backbone.

Table 9 shows that the full model outperformed all ablated variants on both datasets. Two mechanism-level ablations are informative. Removing TR adaptation (No-TR-Adapt) reduced AUC by 1.94 points on ABIDE-I and 1.52 points on ADHD-200. This pattern is consistent with the view that anchoring decay rates to physical time contributes to discrimination. Removing spectral guidance (No-Spec-Guide) led to larger AUC drops (3.08 points on ABIDE-I and 3.32 points on ADHD-200), suggesting that spectral context provides information not fully captured by the temporal backbone alone.

Among single-component ablations, removing SATA was associated with the largest recall reduction (−3.92 points on ABIDE-I), suggesting that spectrum-guided temporal weighting particularly affects sensitivity to positive cases. Removing all three components together produced the largest overall degradation, suggesting that the components provide partially complementary contributions.

**Figure 9:**
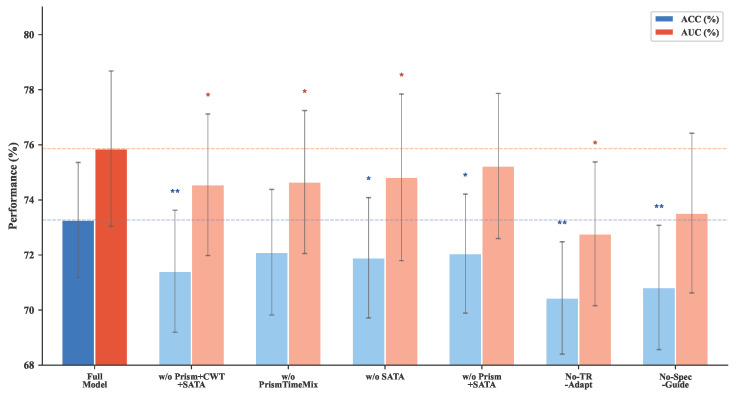
Ablation results on ABIDE-I (Schaefer atlas); error bars denote standard deviation over 50 runs. Removing spectral guidance (No-Spec-Guide) causes the largest single-component drop (−3.08 AUC), and removing all three core components together yields the largest overall degradation. * p<0.05, ** p<0.01 (paired-sample *t*-test versus the full model).

Table 10 reports the paired *t*-test results on ABIDE-I. Figure 9 visualizes the same trend across configurations.

## 4. Discussion

### 4.1. Neurobiological Associations and Interpretability

**Figure 10:**
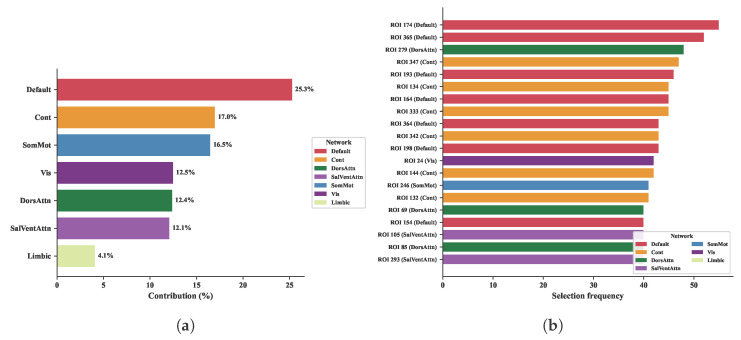
Network-level distribution of model-derived ROI importance on ABIDE-I. (**a**) Proportion of total importance attributed to each Yeo 7-network. (**b**) The 20 highest-ranked individual ROIs and their network affiliations.

We used Gradient-weighted Class Activation Mapping (Grad-CAM) to derive ROI-level importance scores from models trained on the ABIDE-I dataset. As shown in Figure 10, the default mode network accounts for the largest share of total ROI importance (25.3%), followed by the frontoparietal control network (17.0%) and the somatomotor network (16.5%). This ranking is broadly consistent with functional connectivity abnormalities reported in prior ASD neuroimaging studies. The default mode network is implicated in social cognition and self-referential processing, with weakened resting-state connectivity reported in ASD [32] and dysregulated switching with task-positive regions in affected children [33]. The frontoparietal control network, linked to executive function and cognitive flexibility, has shown reduced connectivity associated with repetitive behaviors in ASD [34].

**Figure 11:**
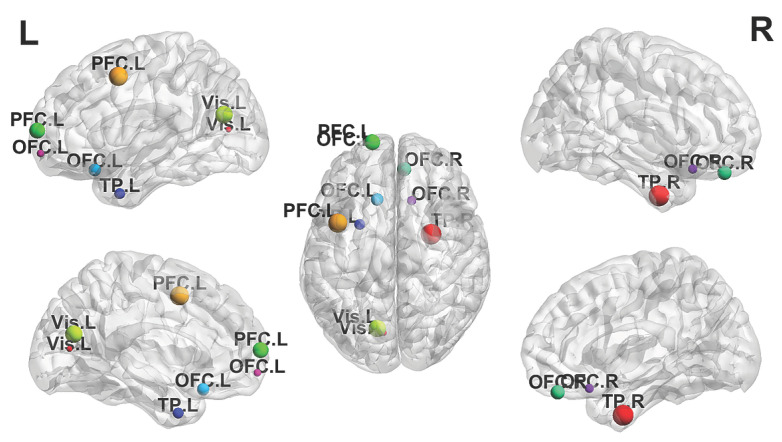
Top 10 ROIs with the largest absolute ASD–TC difference in model-derived importance on ABIDE-I, visualized with BrainNet Viewer [37]. Node size indicates the rank of the absolute difference. Overall, 6 of the 10 ROIs belong to the limbic network, with the temporal pole and orbitofrontal cortex most prominent.

While the network-level analysis identifies overall importance, it does not reveal where the ASD and control groups diverge most. Figure 11 projects the 10 ROIs with the largest absolute group difference (ASD minus TC importance) onto brain space. Six of these ten ROIs belong to the limbic network, concentrated in the temporal pole and orbitofrontal cortex. Altered salience-network connectivity involving the temporal pole has been linked to social motivation deficits in ASD [35], and atypical orbitofrontal–amygdala dynamic connectivity predicts repetitive behavior severity in affected children [36]. The remaining ROIs fall in the default mode network and visual cortex, consistent with the network-level analysis. These two views are complementary: the default mode network carries high overall importance, whereas limbic regions show the largest group differences. This pattern is consistent with the possibility that default mode features contribute broadly across groups, while limbic features are more group-discriminative in the learned importance maps.

**Figure 12:**
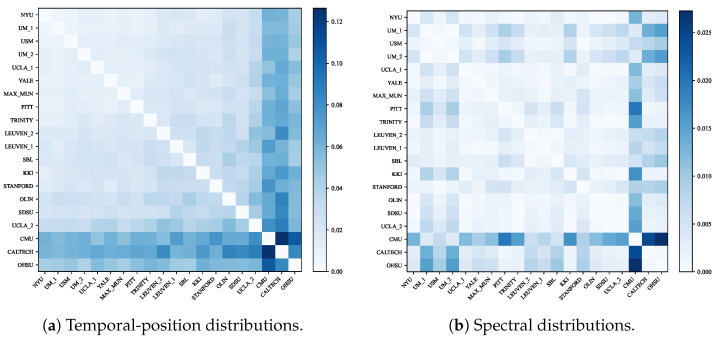
Pairwise Jensen–Shannon divergence of site-level distributions in ABIDE-I. (**a**) High-weight temporal positions. (**b**) Spectral energy distributions. Most site pairs show low divergence.

**Figure A1:**
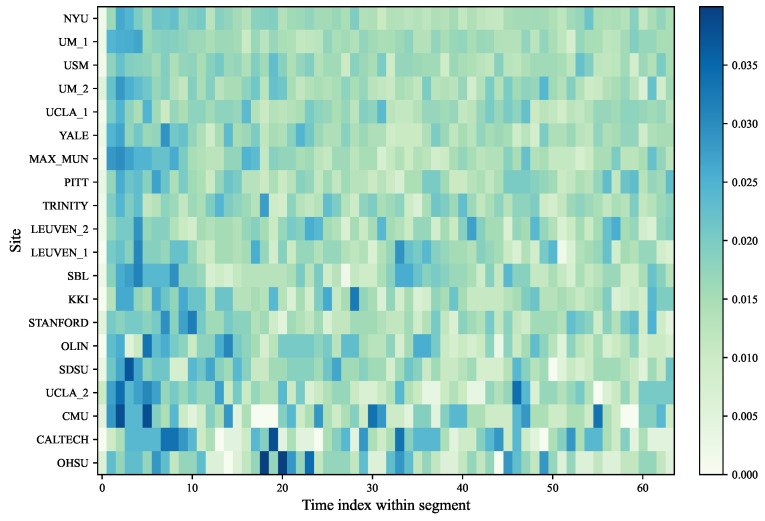
Per-site distribution of high-weight temporal positions under SATA on ABIDE-I. Each panel shows one held-out site. Most sites share a similar concentration pattern; larger deviations are confined to smaller sites.

**Figure A2:**
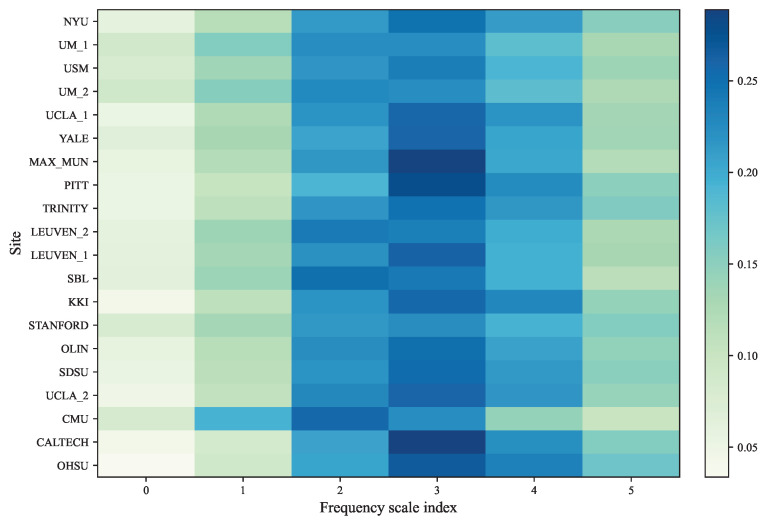
Per-site spectral energy distributions learned by the TR-adaptive spectral branch on ABIDE-I. The dominant energy is concentrated in similar scale ranges across most sites, consistent with the low pairwise JSD values in the main text.

To assess whether the model exploits site-specific artifacts, we computed pairwise Jensen–Shannon divergence (JSD) between sites for both temporal-position and spectral distributions. As shown in Figure 12, most site pairs show low divergence in both domains, indicating that the temporal positions emphasized by SATA and the spectral representations produced by the TR-adaptive branch are broadly shared across sites rather than driven by acquisition differences. The underlying per-site distributions are provided in Appendix A (Figure A1 and Figure A2).

Spectral analysis reveals that the ASD group exhibits higher energy in the slow-4 band (0.027–0.073 Hz) and lower energy in the slow-2 band (0.198–0.25 Hz) compared with controls, consistent with prior reports of altered low-frequency amplitude in ASD [32,38]. The slow-4 band spans periods of roughly 14–37 s, overlapping with typical default mode oscillation time scales, and the elevated energy may reflect altered default mode dynamics during rest.

**Figure 13:**
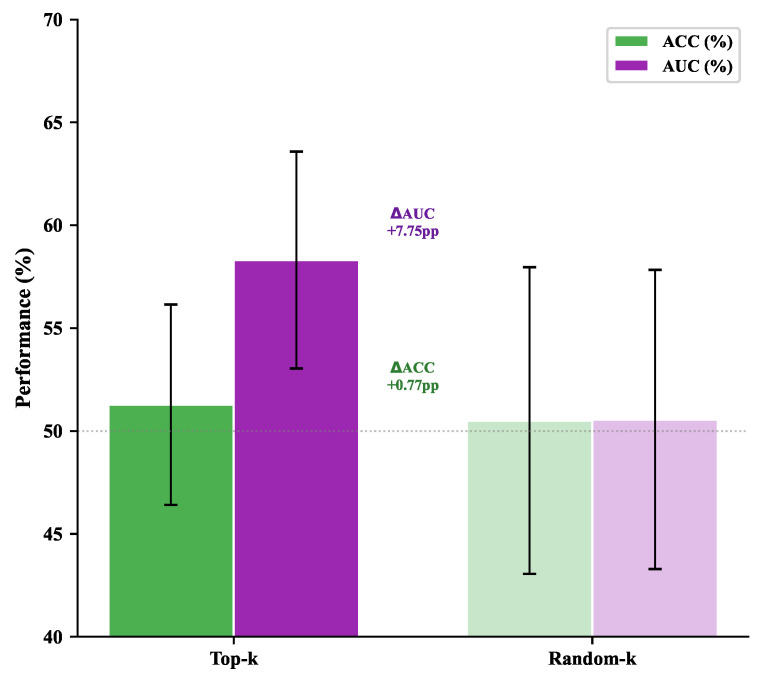
AUC of logistic regression trained on the top 10% SATA-weighted time steps versus randomly selected time steps on ABIDE-I; shade intensity carries no additional meaning.

As a sanity check, we extracted ROI activations from the top 10% of SATA-weighted time steps and trained a logistic regression classifier, comparing against randomly selected time steps. SATA-selected features yield an AUC of 58.31±5.27 versus 50.56±7.27 for the random baseline (Figure 13). The absolute performance is modest because logistic regression operates on single-step activations without temporal context; the relevant finding is the relative gap, which confirms that SATA-selected positions carry more discriminative information than arbitrary time steps.

These analyses suggest partial alignment between the model’s learned representations and disorder-relevant neural systems. Overall importance concentrates on the default mode and frontoparietal networks. Group-discriminative importance localizes to limbic regions. Spectral signatures are consistent with known low-frequency alterations in ASD. Cross-site patterns are broadly shared. These findings indicate neurobiological relevance, though they do not constitute direct evidence of specific neural mechanisms.

### 4.2. Limitations and Future Directions

Several limitations should be acknowledged. The current evaluation covers two public datasets and two parcellation schemes. Although the results demonstrate robustness to TR-related site effects, other sources of cross-site heterogeneity were not systematically examined. These include scanner and vendor differences, acquisition parameters beyond TR (e.g., spatial resolution, echo time), population composition (e.g., age, sex, medication status), and preprocessing variation. This is not unique to our study; prior multi-site rs-fMRI work has shown that such confounds can rival disease effect sizes and remain an open challenge for the field [5].

Second, the present study does not provide causal evidence for the neurobiological relevance of the learned representations. On the data side, the study uses rs-fMRI only, lacks longitudinal follow-up, and relies on coarse diagnostic labels (e.g., ADHD subtypes collapsed into a single category). On the method side, Grad-CAM attributions are gradient-based approximations that do not directly measure neural activity. More broadly, saliency-based explanations do not by themselves establish mechanistic validity, and the spatial overlap with known disorder-related regions, while encouraging, should be interpreted with caution.

Future work should address these gaps along several lines. First, evaluating the framework under explicit harmonization benchmarks and across a wider range of acquisition protocols would strengthen generalizability claims. Second, integrating complementary modalities such as structural MRI, diffusion MRI, and genetic covariates may capture aspects of disorder neurobiology that rs-fMRI alone cannot. Third, adopting causal inference frameworks would allow more rigorous assessment of whether learned features reflect disorder-related mechanisms rather than statistical associations. Finally, extending the approach to longitudinal cohorts and additional neuropsychiatric conditions would help clarify its clinical applicability beyond the cross-sectional binary classification settings studied here.

## 5. Conclusions

This study presents Spec-RWKV, a spectrum-guided linear recurrent framework designed for two challenges in multi-site rs-fMRI-assisted diagnosis: cross-site TR heterogeneity and BOLD signal non-stationarity.

The framework introduces three coordinated mechanisms. PrismTimeMix defines multi-scale temporal decay rates as physical half-lives and converts them per step using each sample’s TR. A TR-adaptive continuous wavelet transform aligns spectral features across sites by adjusting frequency coverage to the local sampling rate. Spectrum-guided adaptive temporal aggregation then uses these spectral features to reweight temporal evidence before classification.

Experiments on ABIDE-I and ADHD-200 show competitive diagnostic performance and improved cross-site generalization relative to the evaluated baselines. Interpretability analyses suggest that the learned representations emphasize the default mode and frontoparietal networks, while spectral features concentrate in the slow-4 band in patterns broadly consistent with prior neuroimaging findings. These results support spectrum-guided recurrent modeling as a promising direction for more comparable cross-site analysis with interpretable intermediate patterns.

## Figures and Tables

**Table 1:** Site-level sample sizes, diagnostic group counts, and repetition times (TR) for the ABIDE-I dataset after quality control (871 subjects across 20 sites).

Site	Subjects	ASD	Controls	TR (s)
NYU	172	74	98	2.0
UM_1	86	34	52	2.0
USM	67	43	24	2.0
UCLA_1	64	37	27	3.0
PITT	50	24	26	1.5
MAX_MUN	46	19	27	3.0
TRINITY	44	19	25	2.0
YALE	41	22	19	2.0
UM_2	34	13	21	2.0
KKI	33	12	21	2.5
OLIN	28	14	14	1.5
LEUVEN_1	28	14	14	∼1.667
LEUVEN_2	28	12	16	∼1.651
SDSU	27	8	19	2.0
SBL	26	12	14	2.2
STANFORD	25	12	13	2.0
OHSU	25	12	13	2.5
UCLA_2	21	11	10	3.0
CALTECH	15	5	10	2.0
CMU	11	6	5	1.5/2.0
Total	871	403	468	

**Table 2:** Site-level sample sizes, diagnostic group counts, and TR values for the ADHD-200 dataset after quality control (635 subjects across 9 sites). Pittsburgh and WashU contain only control subjects.

Site	Subjects	ADHD	Controls	TR (s)
NYU	179	91	88	2.0
Peking_1	78	20	58	2.0
Peking_2	56	33	23	2.0
Peking_3	40	18	22	2.0
KKI	72	18	54	2.5
Pittsburgh	63	0	63	1.5
OHSU	57	22	35	2.5
NeuroIMAGE	39	17	22	1.96
WashU	51	0	51	2.5
Total	635	219	416	

**Table 3:** Mixed-site 10-fold cross-validation results on the Schaefer 400-ROI atlas. Baselines are grouped by modeling strategy: traditional ML, recurrent, graph-based, Transformer-based, and prompt-based. Spec-RWKV achieves the best AUC on both datasets.

Method	ABIDE-I (871 Subjects, 20 Sites)				ADHD-200 (635 Subjects, 9 Sites)			
	ACC (%)	Prec. (%)	Rec. (%)	AUC (%)	ACC (%)	Prec. (%)	Rec. (%)	AUC (%)
SVM	65.43 ± 4.53	65.16 ± 4.50	63.43 ± 4.57	71.13 ± 6.26	65.45 ± 8.19	65.55 ± 5.68	**79.61 ± 7.18**	57.61 ± 6.94
LSTM	54.14 ± 5.63	56.66 ± 5.04	58.91 ± 7.52	55.46 ± 7.27	58.58 ± 4.67	66.39 ± 3.26	74.68 ± 5.62	52.36 ± 7.10
CNN-LSTM	52.11 ± 5.25	54.56 ± 4.56	62.10 ± 7.65	52.26 ± 7.26	53.32 ± 6.07	65.00 ± 4.66	62.14 ± 8.75	50.09 ± 7.65
AttnLSTM	71.86 ± 3.09	72.64 ± 4.46	74.38 ± 6.38	74.15 ± 3.42	71.34 ± 3.22	74.63 ± 3.46	78.13 ± 5.19	71.81 ± 3.57
BrainNetCNN	62.75 ± 6.45	68.22 ± 8.65	54.54 ± 22.48	71.20 ± 6.15	62.42 ± 5.11	69.07 ± 3.73	78.15 ± 8.78	60.08 ± 6.12
PLSNet	72.55 ± 2.73	71.50 ± 4.80	68.14 ± 8.19	75.11 ± 3.56	70.58 ± 3.11	71.86 ± 2.62	72.92 ± 4.81	75.40 ± 2.73
MAHGCN	73.12 ± 3.63	71.05 ± 5.38	72.02 ± 4.14	72.07 ± 3.03	70.76 ± 4.63	69.95 ± 3.38	71.08 ± 2.14	74.25 ± 3.41
SwinT	69.83 ± 4.62	60.68 ± 7.55	68.34 ± 6.49	74.21 ± 4.18	68.49 ± 4.69	65.32 ± 4.41	66.37 ± 9.63	73.92 ± 4.63
BolT	71.28 ± 4.62	69.85 ± 4.94	71.32 ± 4.35	75.14 ± 3.44	69.63 ± 4.63	68.60 ± 5.19	76.56 ± 5.79	72.56 ± 5.02
STARFormer	71.81 ± 4.06	72.48 ± 4.91	**83.25 ± 6.55**	74.34 ± 5.90	71.51 ± 5.69	72.90 ± 5.06	73.74 ± 3.13	74.02 ± 9.01
Com-BrainTF	72.81 ± 4.49	70.58 ± 4.59	78.37 ± 4.70	73.86 ± 3.08	68.88 ± 2.73	67.42 ± 4.18	72.97 ± 3.82	73.47 ± 3.74
BrainPrompt	72.53 ± 3.78	73.85 ± 4.70	75.61 ± 6.55	74.92 ± 3.64	72.12 ± 3.49	**76.24 ± 3.58**	**79.70 ± 5.87**	72.45 ± 3.81
Spec-RWKV	**73.27 ± 4.18**	**74.97 ± 6.70**	77.04 ± 6.41	**75.86 ± 5.64**	**72.87 ± 2.46**	74.64 ± 2.08	70.18 ± 3.17	**76.31 ± 2.51**

Note: Bold values indicate the best-performing results for each metric.

**Table 4:** Comparison with baseline methods on the AAL atlas. The ranking remains broadly consistent with the Schaefer results, indicating stable performance across parcellation schemes.

Method	ABIDE-I (871 Subjects, 20 Sites)				ADHD-200 (635 Subjects, 9 Sites)			
	ACC (%)	Prec. (%)	Rec. (%)	AUC (%)	ACC (%)	Prec. (%)	Rec. (%)	AUC (%)
SVM	63.74 ± 3.99	52.35 ± 7.18	63.60 ± 6.35	69.90 ± 4.53	57.96 ± 2.60	56.96 ± 5.16	57.82 ± 5.16	59.40 ± 4.52
LSTM	64.77 ± 4.12	62.12 ± 9.73	63.17 ± 7.24	68.84 ± 3.77	62.10 ± 3.84	60.69 ± 4.17	58.66 ± 6.06	64.24 ± 5.40
CNN-LSTM	64.49 ± 5.70	57.31 ± 9.40	64.79 ± 7.57	71.40 ± 5.41	63.58 ± 5.82	58.24 ± 9.33	61.68 ± 7.44	60.37 ± 5.29
AttnLSTM	72.56 ± 3.28	71.89 ± 4.12	75.62 ± 5.61	74.68 ± 3.35	73.29 ± 3.14	68.90 ± 3.85	69.43 ± 4.93	76.52 ± 3.22
BrainNetCNN	65.31 ± 4.09	60.90 ± 8.56	62.88 ± 4.40	70.68 ± 5.51	61.77 ± 3.78	60.11 ± 4.52	60.63 ± 7.91	60.77 ± 4.01
PLSNet	71.96 ± 3.38	69.87 ± 3.47	75.69 ± 3.57	74.43 ± 3.12	70.53 ± 3.14	71.81 ± 2.03	72.97 ± 4.86	75.45 ± 2.78
MAHGCN	71.31 ± 3.52	69.40 ± 3.27	70.09 ± 3.93	71.11 ± 2.97	69.69 ± 2.49	71.36 ± 3.28	70.19 ± 4.23	71.86 ± 3.31
SwinT	65.69 ± 4.45	58.92 ± 7.45	60.53 ± 0.53	72.64 ± 4.07	66.50 ± 4.60	62.64 ± 4.23	65.22 ± 9.22	66.29 ± 4.45
BolT	69.41 ± 2.15	68.52 ± 4.07	66.49 ± 4.22	73.30 ± 3.51	67.66 ± 3.46	68.15 ± 4.05	68.36 ± 5.01	69.83 ± 3.74
STARFormer	73.11 ± 2.89	72.96 ± 2.19	78.31 ± 3.47	74.01 ± 2.79	72.92 ± 2.40	72.59 ± 2.02	73.12 ± 3.23	76.39 ± 2.45
Com-BrainTF	70.56 ± 4.42	68.53 ± 4.41	77.21 ± 4.25	75.37 ± 2.96	66.87 ± 2.60	69.74 ± 4.10	70.73 ± 3.59	72.06 ± 3.51
BrainPrompt	73.38 ± 3.52	72.78 ± 3.85	76.84 ± 5.42	75.47 ± 3.18	73.99 ± 3.26	70.03 ± 3.72	70.57 ± 5.15	77.70 ± 3.08
Spec-RWKV	**75.19 ± 2.93**	**73.96 ± 2.13**	**79.27 ± 3.42**	**75.91 ± 2.85**	**74.98 ± 2.36**	**74.81 ± 2.04**	**75.31 ± 3.19**	**78.47 ± 2.50**

Note: Bold values indicate the best-performing results for each metric.

**Table 5:** Leave-one-site-out (LOSO) results, where one site is held out entirely for testing and the model is trained on the remaining sites. Standard deviations reflect variation across held-out sites. On ABIDE-I, Spec-RWKV achieves the best aggregate AUC among the evaluated methods. Compared with mixed-site evaluation (Table 3), this stricter protocol remains consistent with a role for TR-aware modeling when the test site is entirely unseen.

Method	ABIDE-I (20 Sites)				ADHD-200 (9 Sites)			
	ACC (%)	Prec. (%)	Rec. (%)	AUC (%)	ACC (%)	Prec. (%)	Rec. (%)	AUC (%)
SVM	60.65 ± 9.29	62.27 ± 10.98	73.26 ± 17.85	65.49 ± 10.26	56.33 ± 7.22	60.85 ± 10.44	**70.97 ± 15.74**	57.18 ± 6.80
BrainNetCNN	62.73 ± 10.86	66.48 ± 18.22	65.57 ± 23.91	69.68 ± 11.97	52.77 ± 9.24	59.41 ± 11.08	60.86 ± 19.86	53.97 ± 5.20
SwinT	63.74 ± 14.32	58.59 ± 17.41	69.61 ± 10.61	69.75 ± 13.91	52.74 ± 6.65	55.09 ± 10.72	54.56 ± 10.42	53.41 ± 6.58
BolT	67.39 ± 10.58	68.13 ± 12.68	76.51 ± 18.09	70.24 ± 12.64	55.31 ± 7.94	60.35 ± 12.19	64.07 ± 15.64	58.51 ± 6.08
PLSNet	69.84 ± 13.28	69.44 ± 13.45	86.04 ± 14.10	71.44 ± 13.00	56.00 ± 4.49	63.23 ± 7.79	61.09 ± 14.07	60.79 ± 4.24
STARFormer	70.87 ± 12.80	72.58 ± 12.18	**89.05 ± 13.99**	71.06 ± 12.68	57.91 ± 8.90	63.83 ± 9.78	61.19 ± 12.70	61.63 ± 5.97
AttnLSTM	71.38 ± 10.42	75.14 ± 11.38	74.86 ± 14.17	73.52 ± 10.65	60.42 ± 9.12	69.87 ± 10.24	63.71 ± 13.56	64.38 ± 7.89
BrainPrompt	72.05 ± 9.86	76.82 ± 10.24	75.43 ± 13.52	74.63 ± 9.87	61.70 ± 8.74	**71.19 ± 9.56**	**65.03 ± 12.84**	65.84 ± 7.23
Spec-RWKV	**72.78 ± 8.52**	**79.05 ± 7.53**	72.22 ± 11.43	**75.86 ± 9.21**	**65.70 ± 8.58**	69.94 ± 8.12	60.26 ± 9.71	**67.13 ± 5.57**

Note: Bold values indicate the best-performing results for each metric.

**Table 6:** Summary of per-site LOSO performance on ABIDE-I. BA, balanced accuracy; IQR, interquartile range. A higher mean BA with a lower spread indicates better cross-site stability.

Method	BA Mean	BA Std	95% CI	AUC Mean	AUC Std	95% CI	IQR
Spec-RWKV	**0.7366**	0.0843	[0.697, 0.776]	0.7186	0.1274	[0.659, 0.778]	**0.078**
BolT	0.6661	0.0959	[0.621, 0.711]	**0.7424**	0.1211	[0.686, 0.799]	0.120
BrainPrompt	0.6892	0.1086	[0.637, 0.741]	0.7156	0.1124	[0.662, 0.769]	0.128
BrainNetCNN	0.6331	0.0761	[0.598, 0.669]	0.6968	0.1065	[0.647, 0.747]	0.103
AttnLSTM	0.6438	0.1012	[0.596, 0.691]	0.6724	0.1385	[0.607, 0.738]	0.122
SVM	0.5990	0.1083	[0.548, 0.650]	0.6549	0.1017	[0.607, 0.703]	0.099

Note: Bold values indicate the best-performing results for each metric.

**Table 7:** Site prediction from learned embeddings on ABIDE-I. Lower balanced accuracy indicates weaker site-discriminative information in the representation.

Method	Dim.	Site BA	Chance	BA/Chance	*p*-Value
AttnLSTM	256	0.0823	0.05	1.65×	0.0010
Spec-RWKV	256	0.1238	0.05	2.48×	0.005
BolT	2400	0.2158	0.05	4.32×	0.005
BrainPrompt	256	0.3412	0.05	6.82×	0.005

**Table 8:** Output stability under simulated TR resampling on ABIDE-I. Time series were resampled to multiple target TR values via piecewise linear interpolation and re-evaluated with trained models. Because the evaluation set includes training samples, absolute AUC values should not be compared with the held-out results in Table 3; the focus is on relative stability across TR conditions.

Simulated TR	Spec-RWKV AUC	BrainPrompt AUC	AttnLSTM AUC
1.5 s	0.9716	0.9178	0.9356
2.0 s	0.9787	0.9135	0.9382
2.5 s	0.9815	0.9087	0.9408
3.0 s	0.9829	0.8996	0.9271
4.0 s	0.9817	0.9124	0.9098

**Table 9:** Ablation study results on ABIDE-I and ADHD-200. The full model achieves the best performance across both datasets.

Configuration	ABIDE-I				ADHD-200			
	ACC (%)	Prec. (%)	Rec. (%)	AUC (%)	ACC (%)	Prec. (%)	Rec. (%)	AUC (%)
**Full Model**	**73.27 ± 4.18**	**74.97 ± 6.70**	**77.04 ± 6.41**	**75.86 ± 5.64**	**72.87 ± 2.46**	**74.64 ± 2.08**	70.18 ± 3.17	**76.31 ± 2.51**
w/o Prism+CWT+SATA	71.41 ± 4.43	73.95 ± 6.85	74.15 ± 7.17	74.55 ± 5.14	65.85 ± 3.49	69.20 ± 4.49	71.03 ± 9.87	66.37 ± 4.89
w/o PrismTimeMix	72.10 ± 4.56	74.09 ± 5.67	74.80 ± 6.56	74.65 ± 5.19	66.51 ± 3.60	69.53 ± 3.69	72.28 ± 10.06	67.17 ± 4.30
w/o SATA	71.90 ± 4.37	74.93 ± 6.15	73.12 ± 6.10	74.82 ± 6.05	65.02 ± 3.12	69.66 ± 4.45	66.00 ± 12.08	66.57 ± 3.70
w/o Prism+SATA	72.05 ± 4.32	73.40 ± 6.18	76.74 ± 6.36	75.23 ± 5.27	65.35 ± 3.34	68.79 ± 4.05	69.70 ± 11.18	66.38 ± 4.61
No-TR-Adapt	71.56 ± 3.45	72.13 ± 4.82	75.68 ± 7.12	73.92 ± 3.71	71.36 ± 3.01	72.11 ± 4.23	68.86 ± 8.64	74.79 ± 3.39
No-Spec-Guide	70.89 ± 3.56	71.56 ± 4.95	74.21 ± 7.34	72.78 ± 3.83	70.51 ± 3.10	70.83 ± 4.35	67.69 ± 8.91	72.99 ± 3.50

Note: Bold values indicate the best-performing results for each metric.

**Table 10:** Paired *t*-test results for ablation studies on ABIDE-I. Δ represents the improvement of the full model over each ablation configuration.

Comparison	Metric	Δ (pp)	t-Statistic	*p*-Value	Sig.
w/o Prism + CWT + SATA	ACC	+1.86	2.993	0.004	**
	AUC	+1.31	2.112	0.040	*
	Recall	+2.89	2.183	0.034	*
w/o PrismTimeMix	ACC	+1.17	1.998	0.051	
	AUC	+1.21	2.064	0.044	*
	Recall	+2.24	2.147	0.037	*
w/o SATA	ACC	+1.38	2.604	0.012	*
	AUC	+1.04	2.231	0.030	*
	Recall	+3.92	2.876	0.006	**
w/o Prism + SATA	ACC	+1.22	2.366	0.022	*
	AUC	+0.63	1.048	0.300	
	Recall	+0.30	0.429	0.670	
No-TR-Adapt	ACC	+1.71	3.866	0.004	**
	AUC	+1.94	2.508	0.033	*
	Recall	+1.36	1.469	0.176	
No-Spec-Guide	ACC	+2.38	3.386	0.008	**
	AUC	+3.08	1.619	0.140	
	Recall	+2.83	1.160	0.276	

Note: Δ represents improvement of the full model over ablation configuration (percentage points); * p<0.05, ** p<0.01.

## Data Availability

The data presented in this study are openly available in ABIDE Preprocessed Connectomes Project at http://preprocessed-connectomes-project.org/abide/ (accessed on 21 April 2026) and ADHD-200 Preprocessed Connectomes Project at http://preprocessed-connectomes-project.org/adhd200/ (accessed on 21 April 2026). The code for Spec-RWKV is available at https://github.com/arisaris28/spec-rwkv (accessed on 26 March 2026).

## Appendix A. Additional Experimental Results

### Appendix A.1. Per-Site Leave-One-Site-Out Performance

Table A1 reports the per-site LOSO results of Spec-RWKV on ABIDE-I under the Schaefer 400-ROI atlas. Each row corresponds to one held-out site. Balanced accuracy (BA) and AUC are averaged over five random seeds; *n* is the number of test subjects when that site is held out.

**Table A1:** Per-site LOSO performance of Spec-RWKV on ABIDE-I (Schaefer 400-ROI atlas). BA, balanced accuracy. Values are mean ± std over 5 seeds.

Site	*n*	BA	AUC	Sensitivity	Specificity	Macro-F1
CALTECH	7	0.920 ± 0.110	0.900 ± 0.141	0.840 ± 0.219	1.000 ± 0.000	0.883 ± 0.160
CMU	7	0.838 ± 0.106	0.778 ± 0.157	0.867 ± 0.183	0.808 ± 0.248	0.836 ± 0.117
KKI	32	0.757 ± 0.027	0.820 ± 0.031	0.780 ± 0.130	0.733 ± 0.109	0.749 ± 0.042
LEUVEN_1	11	0.564 ± 0.051	0.314 ± 0.089	0.700 ± 0.411	0.429 ± 0.404	0.458 ± 0.120
LEUVEN_2	17	0.790 ± 0.037	0.825 ± 0.023	0.756 ± 0.145	0.825 ± 0.190	0.783 ± 0.036
MAX_MUN	44	0.596 ± 0.082	0.536 ± 0.097	0.533 ± 0.268	0.659 ± 0.210	0.560 ± 0.114
NYU	165	0.647 ± 0.084	0.683 ± 0.099	0.766 ± 0.177	0.528 ± 0.316	0.615 ± 0.143
OHSU	25	0.670 ± 0.069	0.628 ± 0.085	0.523 ± 0.280	0.817 ± 0.231	0.637 ± 0.102
OLIN	28	0.736 ± 0.086	0.726 ± 0.097	0.929 ± 0.101	0.543 ± 0.120	0.724 ± 0.091
PITT	49	0.739 ± 0.026	0.753 ± 0.022	0.744 ± 0.067	0.733 ± 0.048	0.738 ± 0.027
SBL	20	0.724 ± 0.056	0.673 ± 0.098	0.582 ± 0.104	0.867 ± 0.122	0.706 ± 0.056
SDSU	14	0.775 ± 0.056	0.755 ± 0.057	0.600 ± 0.158	0.950 ± 0.112	0.690 ± 0.084
STANFORD	25	0.735 ± 0.034	0.721 ± 0.032	0.569 ± 0.140	0.900 ± 0.149	0.718 ± 0.040
TRINITY	38	0.651 ± 0.099	0.632 ± 0.107	0.667 ± 0.218	0.635 ± 0.378	0.610 ± 0.151
UCLA_1	62	0.742 ± 0.050	0.752 ± 0.029	0.840 ± 0.063	0.643 ± 0.107	0.721 ± 0.058
UCLA_2	21	0.816 ± 0.039	0.762 ± 0.046	0.760 ± 0.055	0.873 ± 0.081	0.817 ± 0.040
UM_1	85	0.713 ± 0.056	0.744 ± 0.064	0.681 ± 0.199	0.746 ± 0.170	0.694 ± 0.071
UM_2	34	0.718 ± 0.065	0.705 ± 0.049	0.743 ± 0.164	0.692 ± 0.255	0.701 ± 0.054
USM	65	0.776 ± 0.030	0.813 ± 0.023	0.733 ± 0.109	0.820 ± 0.086	0.773 ± 0.029
YALE	37	0.827 ± 0.024	0.856 ± 0.030	0.833 ± 0.039	0.821 ± 0.047	0.827 ± 0.024

**Table A2:** Per-site LOSO performance of Spec-RWKV on ADHD-200 for selected sites (excluding Pittsburgh and WashU, which contain only control subjects). BA, balanced accuracy. Values are mean ± std over 5 seeds.

Site	*n*	BA	AUC	Sensitivity	Specificity	Macro-F1
NYU	179	0.550 ± 0.029	0.617 ± 0.043	0.476 ± 0.034	0.624 ± 0.049	0.532 ± 0.020
Peking_1	78	0.691 ± 0.065	0.696 ± 0.062	0.634 ± 0.058	0.748 ± 0.079	0.678 ± 0.071
Peking_2	56	0.666 ± 0.070	0.682 ± 0.059	0.606 ± 0.098	0.725 ± 0.070	0.653 ± 0.080
Peking_3	40	0.646 ± 0.079	0.671 ± 0.074	0.586 ± 0.106	0.706 ± 0.119	0.631 ± 0.076
OHSU	57	0.658 ± 0.053	0.683 ± 0.055	0.605 ± 0.092	0.710 ± 0.096	0.652 ± 0.060
KKI	72	0.781 ± 0.046	0.754 ± 0.074	0.746 ± 0.060	0.817 ± 0.066	0.781 ± 0.051
NeuroIMAGE	39	0.747 ± 0.065	0.735 ± 0.091	0.706 ± 0.120	0.789 ± 0.094	0.743 ± 0.080

### Appendix A.2. Per-Site Cross-Site Consistency

To complement the pairwise JSD matrices in the main text (Figure 12), Figure A1 and Figure A2 show the per-site distributions from which those divergence values were computed.
